# Female genital tuberculosis and infertility: serial cases report in Bandung, Indonesia and literature review

**DOI:** 10.1186/s13104-017-3057-z

**Published:** 2017-12-04

**Authors:** Tono Djuwantono, Wiryawan Permadi, Leri Septiani, Ahmad Faried, Danny Halim, Ida Parwati

**Affiliations:** 1 Department of Obstetrics and Gynecology, Faculty of Medicine, Universitas Padjadjaran–Dr. Hasan Sadikin Hospital, Bandung, Indonesia; 2 Oncology and Stem Cell Working Group, Faculty of Medicine, Universitas Padjadjaran–Dr. Hasan Sadikin Hospital, Bandung, Indonesia; 3 Department of Clinical Pathology, Faculty of Medicine, Universitas Padjadjaran–Dr. Hasan Sadikin Hospital, Bandung, Indonesia; 4 Faculty of Medicine, Universitas Padjadjaran–Dr. Hasan Sadikin Hospital, Jl. Pasteur No. 38, Bandung, 40161 West Java Indonesia

**Keywords:** Serial cases report, Female genital tuberculosis, Infertility, Laparoscopy, Histopathology examination, Polymerase chain reaction

## Abstract

**Background:**

Female genital tuberculosis (FGTB) is a Mycobacterium infection in the reproductive organs which often leads to infertility. FGTB is either asymptomatic or causes uncharacteristic clinical presentations, making an early diagnosis is challenging. Our aims were to evaluate the clinical presentations, the process to confirm the diagnosis and followed-up the patients who had undergone laparoscopy at our center. FGTB has been reported from many countries, but has never been reported from Indonesia. Here we present case studies to document the presence of FGTB in Indonesia.

**Cases presentation:**

There were three patients admitted to our center; two patients were admitted with irregular menstrual cycle as their chief complaint, while one patient came due to infertility. The results from laparoscopy were suggestive of FGTB; including the presence of caseating granulomas surrounded by epithelioid cells, lymphocytes, plasma cells, and Langhans giant cells. Additionally, PCR testing confirmed presence of MTB. Subsequent to diagnosis, continuous TB medications was administered with excellent clinical outcome in two patients (pregnant in 18 months after under gone laparoscopy). The infertile patient remain in one of the treated patient above.

**Conclusion:**

In infertile patients who live in countries where Tuberculosis is an endemic disease, such as Indonesia, a comprehensive history taking, along with ultrasonography results can be used to diagnose FGTB. Confirmation of this diagnosis can be achieved through polymerase chain reactions result. Timely diagnosis and treatment are imperative to prevent any permanent injury to patient’s reproductive organs.

## Background

Tuberculosis (TB) is a disease caused by Mycobacterium TB. In spite of major improvements in antibiotic regiments and vaccination, TB remains as a major global health problem. Based on WHO’s high burden country list for TB, Indonesia is ranked 2nd after India as a country with the highest prevalence of TB [1]. Most commonly, TB infects the lungs (pulmonary TB). However, extrapulmonary manifestation of TB is an increasingly common feature [2]. In female patients, one of the most common site of extrapulmonary TB is the reproductive organs, termed genital TB [3]. Clinically, female genital tuberculosis (FGTB) is usually presented with chronic pelvic inflammatory disease, menstrual abnormalities and infertility [4]. The actual number of FGTB incidences cannot be estimated accurately, as it is often asymptomatic and only 50% of cases are diagnosed without surgery [5–7]. This study was aims to evaluate the clinical presentations, the roles of surgery, histopathology examination and polymerase chain reaction (PCR) assay in three FGTB cases treated at the Department of Obstetrics and Gynecology, Faculty of Medicine, Universitas Padjadjaran (FK UNPAD)–Dr. Hasan Sadikin, Hospital (RSHS), Bandung.

## Patients and methods

### Study design

This is a cohort study that was conducted by examining the medical records of three FGTB patients who were treated at the Department of Obstetrics and Gynecology, FK UNPAD–RSHS Bandung in 2016. Final diagnosis was made by histopathology findings and PCR assay.

### Clinical samples

Surgical specimens were obtained from three patients who were diagnosed FGTB and underwent potentially curative surgery at our center. The study was conducted on FGTB patients whose tissue specimens and complete post-surgery clinical data were available. Tissue specimens were collected from every case, and were snapped frozen in liquid nitrogen for 10 min before stored at – 80 °C. All specimens were then stained with hematoxylin and eosin (HE), and final examination was performed with light microscopy.

### PCR

Peripheral blood was collected from all three patients and DNA isolation was performed by using standard protocol. DNA samples were amplified using following primers IS6110F: 5′–CCTGCGAGCGTAGGCGTCGG–3′ and IS6110R: 5′–CTCGTCCAGCGCCGCTTCGG–3′, with expected product length of 123 base pairs (bp). PCR assay was performed by using standard protocols as described previously [8].

## Case presentations

Three cases of FGTB were evaluated in this study, in which irregular menstrual cycle and infertility were presented as chief complaints in two patients, and one patient’s main complaint was infertility despite having a regular menstrual cycle.

### Case 1

A 30 year old nulliparous was referred to with a chief complaint of an irregular menstrual cycle. Patient had been married for 5 years and unable to conceive despite having a regular unprotected intercourse. Ultrasound examination identified a bilateral endometriotic cysts, thus she was immediately scheduled for laparoscopy. During surgery, bilateral pyosalpinx was visible. Patient was treated and discharged 1 week after surgery.

### Case 2

Patient 2 was a 30 years old nulliparous, who was admitted to our fertility clinic with a history of irregular menstrual cycle. Her last menstrual period was 1 year prior to her visit. Patient had been married for 2.5 years and unable to conceive. Based on her anamnesis, patient was diagnosed with secondary amenorrhoea and infertility. Patient underwent laparoscopic and both of the fallopian tubes were severely damaged with adhesions to surrounding tissues. Patient was discharged 1 day after the surgery and referred to internal medicine department for further evaluation.

### Case 3

A 30 years old nulliparous came to our fertility clinic with chief complaint of inability to conceive after 3 years of marriage. Patient had regular menstrual cycle (30–31 days), with no other related complaints. During ultrasound examination, irregularity in uterine mucosa with bilateral tubal obstruction was observed. Patient was diagnosed with endometriotic cysts with bilateral tubal obstruction, thus she was admitted for laparoscopic; on surgery, bilateral salpingectomy was performed due to obstruction at both of the fallopian tubes and adhesion to the surrounding tissues.

## Results

### Histology examination results

**Fig. 1:**
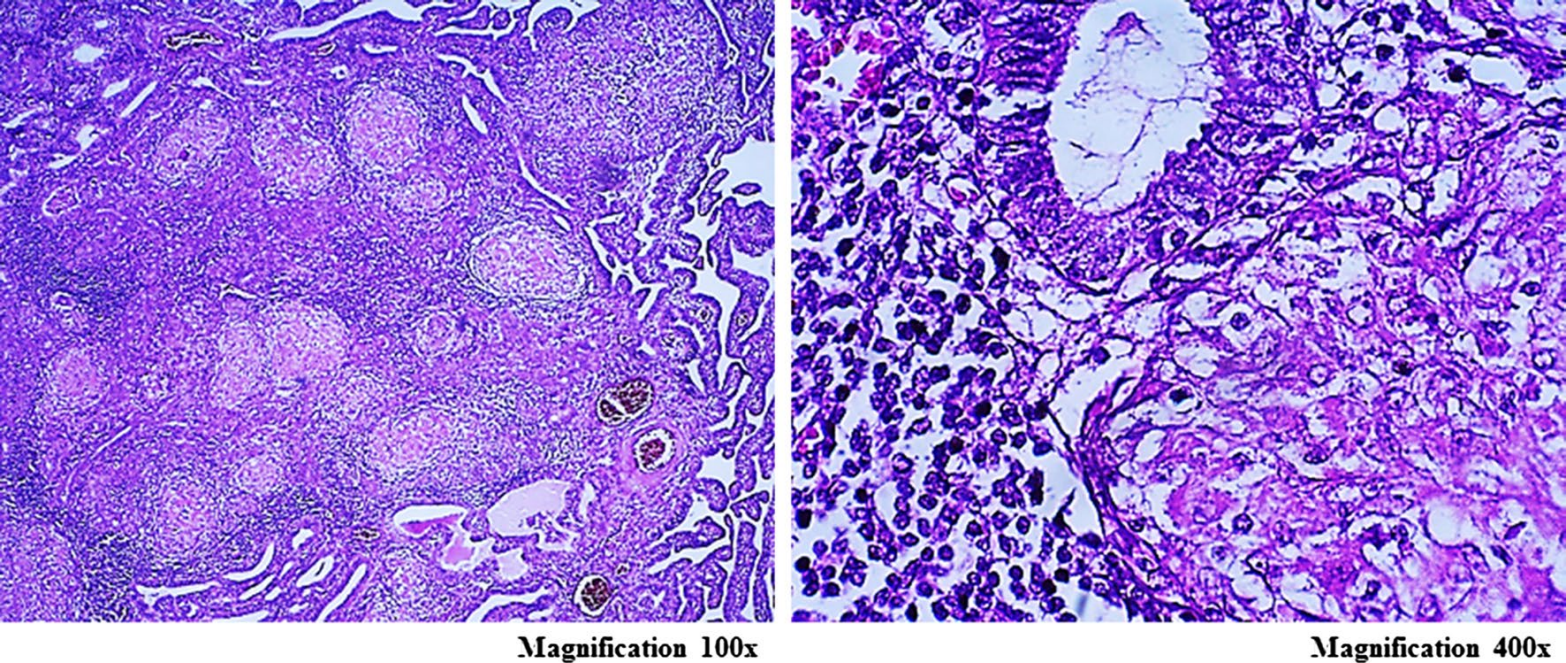
In case 1 the results showed there are tubercles with presence of caseating granulomas surrounded by epithelioid cells, lymphocytes, plasma cells and giant cells (magnification 100× and 400×)

**Fig. 2:**
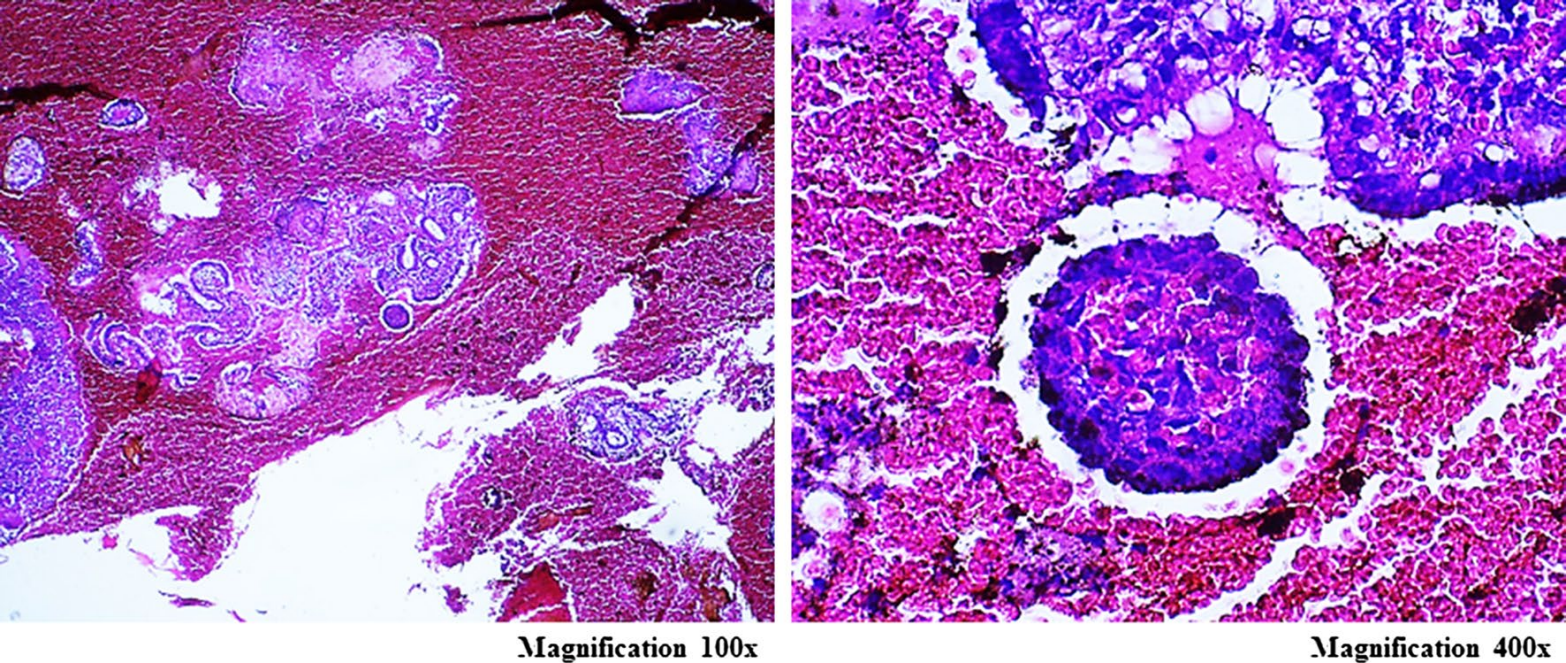
In case 2 the final histopathology finding revealed TB infection that shown as the accumulation of the epitheloid granulomas, datia Langhans cells, and caseous necrosis (magnification 100× and 400×)

**Fig. 3:**
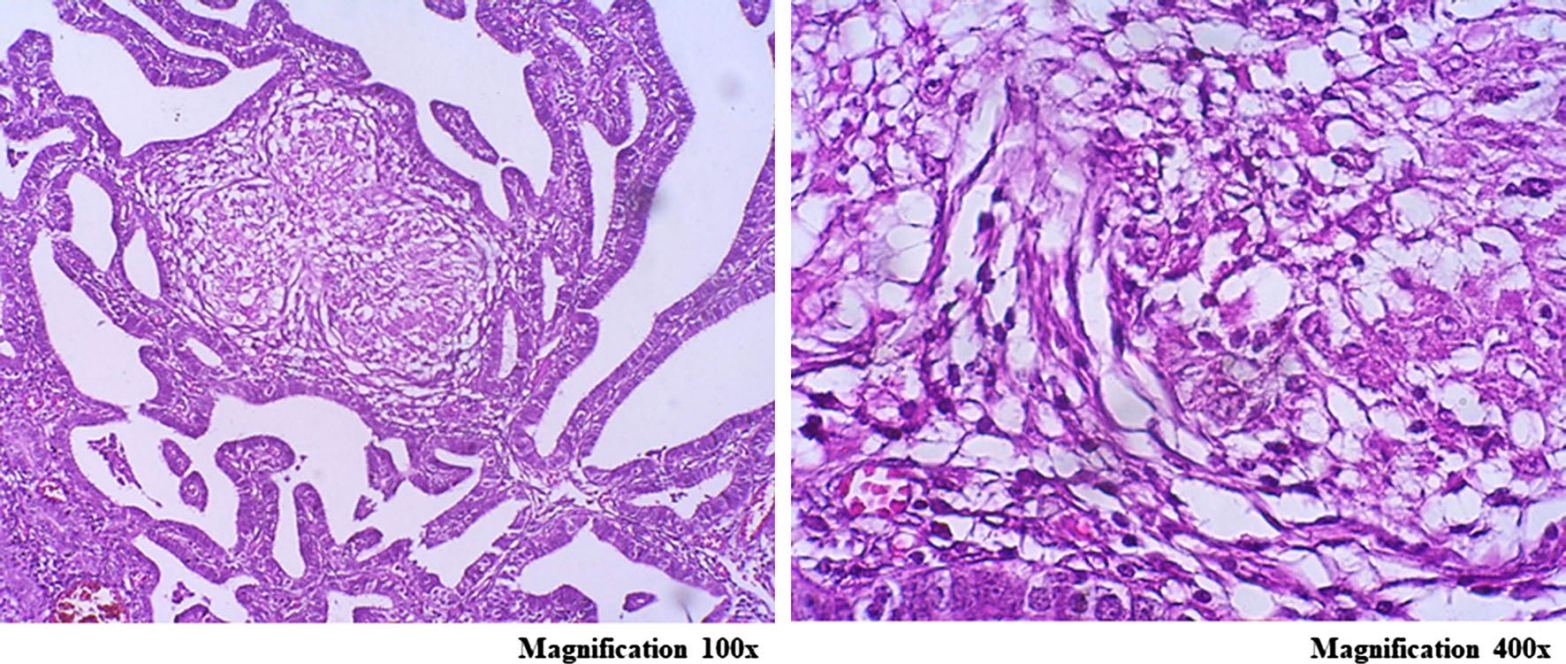
In case 3 photomicrograph showing tuberculous granulomas in the mucosa layer of fallopian tube and fused plica (magnification 100× and 400×)

Specimens were collected from all three patients. Presence of caseating granulomas surrounded by epithelioid cells, lymphocytes, plasma cells and giant cells are diagnostic of GTB [9]. In case 1, the results showed the presence of tubercles with caseating granulomas surrounded by epithelioid cells, lymphocytes, plasma cells and giant cells (Fig. 1). The results from case 2 showed necrotic fallopian tubes tissue, with tubercles and Langhans giant cells (Fig. 2). Therefore, patient 2 was diagnosed with endometrial TB. In case 3, histopathology examination identified the presence of tubercles and Langhans giant cells in the wall of the fallopian tubes. Thus, photomicrograph showing tuberculous granulomas in the mucosa layer of fallopian tube and fused plica (Fig. 3), patient was diagnosed as bilateral TB of the fallopian tubes.

### PCR examination results

**Fig. 4:**
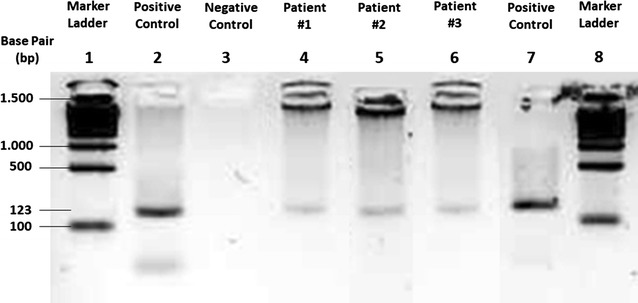
Polymerase chain reaction (PCR) results. The IS6110 primers amplify a fragment of Mycobacterium TB with a length of 123 base pair (bp)

Until now, there is no consensus on tests that should be used as a gold standard in confirming the diagnosis of FGTB. However, it has been commonly accepted that patient who is suspected of having GTB should have some suggestive findings at laparoscopy, with one or more of the following findings: A definite past history of TB, in the presence of active extragenital TB, characteristic features identified through hysterosalpingography, elevated erythrocyte sediment rate, Mantoux test (+), evidence of calcification/complex adnexal mass by scan. Therefore, in this study, we considered patients’ clinical information and laparoscopic evaluation to suspect FGTB. In every patient evaluated in this study, PCR results were positive in all cases (Fig. 4), although the patient’s bands looked weaker when compared with the bands in positive control.

#### Followed-up

Diagnosis of FGTB was made based on pathology and PCR result above; all patients had been treated continuous TB medications with excellent clinical outcome in two patients (pregnant in 18 months after under gone laparoscopy operation), but menstrual abnormalities remains, uterine abnormal bleeding, in one patient.

## Discussion and conclusion

The prevalence of GTB is approximately 27% (range, 14–41%) in worldwide [10]. However, the actual prevalence of GTB cannot be estimated accurately because in some cases, Mycobacterium tuberculosis infection may cause no significant clinical symptoms. In Indonesia, TB is an endemic disease, thus it has been speculated that FGTB is prevalent among Indonesian women [11]. To date, there is no specific data about the incidence of FGTB in Indonesia has ever been reported. Based on the affected organ(s), TB can be classified into two forms: pulmonary and extrapulmonary. GTB is a form of extrapulmonary TB that affects 12.1% of patients with pulmonary TB and represents 15–20% of extra pulmonary TB [12]. It has been estimated that 5–13% of patient in fertility clinics have GTB. Majority of these patients are predicted to be in the age group of 20–40 years old [13]. In most cases, GTB is secondarily acquired by hematogenous spread from an extragenital source, such as pulmonary or abdominal TB. Anatomically, GTB is mainly infested in the fallopian tubes and endometrium, causing infertility as the most likely result [5, 14, 15]. If infection is not recognized early, fulminating destruction of these organs could result in permanent inability to conceive.

### Pathogenesis [16]

GTB is almost always secondary to other TB infections in the body with most common infected sites is lung. Other organs, including bone, joint, gastrointestinal and renal. If patients are not treated well to eradicate the bacteria, there is chance that the bacteria will be reactivated especially when the immune response of the patients is decreased. Getting diseases or drugs that cause attenuation of T cell response (e.g. Hodgkin’s lymphoma, AIDS, steroids, stress or malnutrition) will also increase the risk of bacteria reactivation. The mode of spread is usually hematogenous or lymphatic and occasionally occurs by way of direct contiguity with an intra-abdominal or peritoneal focus. In some cases, treatment usually focus on lung sites but other lesions may lie dormant in other sites such as genital tract and could be reactivate later.

### Hematogenous spread [16]

When tubercle bacilli invade the lung, it will enter into the bloodstream and spread through various organs in the body. This bacterium will remain in the body if not diagnosed and treated with anti TB drugs. In GTB, fallopian tubes is the most common sites for tubercle bacilli infections with the earliest lesion found in the mucosa. Tubercle bacilli infection almost always infected bilateral tubes.

### Lymphatic spread [16]

Infection of tubercle bacilli through lymphatic spread usually happens if the primary lesions originate from abdominal cavity. It is more and less common infection routes.

### Direct spread from a neighboring viscus [16]

Most of the literature state that genital tract never became the primary infection sites. The criteria necessary for a diagnosis of primary GTB are that the genital lesions should be the first TB infection in the body and regional lymph nodes should demonstrate the same stage of TB development as do the genital organs.

### Endometrial TB

Endometrial TB was difficult to diagnose. In women infected with Endometrial TB, it is often asymptomatic or present with non-specific symptoms. The appearance of the disease usually different in women on reproductive age or postmenopausal women. Most common symptoms in reproductive ages of women are menstrual disturbance, uterine abnormal bleeding, or pelvic pain. The menstrual cycle may be normal and undisturbed in some cases of GTB. Superficial tuberculous endometritis does not interfere with the secretory response of the endometrium to hormonal stimulation. When the menstrual cycle is disturbed, some theory stated that it may result from active pulmonary TB that produce amenorrhea, if it is associated with fever and weight loss. But, not all of active pulmonary TB found concomitantly with active GTB. Amenorrhoea from GTB can be could be resulted from to end organ failure secondary to endometrial caseation. Symptoms commonly occur on postmenopausal woman are postmenopausal bleeding, pyometra, or leucorrhoea [14, 17–19].

When extensive involvement of the endometrium occurs, there may be ulcerative, granular, or fungating lesions present, or the endometrial cavity may be obliterated with intrauterine adhesions. Sometimes, the macroscopic appearance may resemble carcinoma, and TB has been suggested microscopically. In some cases, total destruction of the endometrium with resulting amenorrhea secondary to end-organ failure and predisposition to pyometra should the internal is becomes occluded. Imaging is not a gold standard for diagnosing Endometrial TB but it may help to confirm the diagnosis. A thickened endometrium or pyometra can be found during transvaginal ultrasound. A distorted contour of uterine cavity commonly found during hysterosalpingogram procedure. Endometrial biopsy is a mandatory procedure to confirm the typical non-caseating lesion consistent with TB [18].

### Tuberculosis of the fallopian tubes and infertility

Infertility defined as failure of a couple to get offspring after 1 year (< 35 years old) or 6 months (> 35 years old) with regular sexual intercourse (3–4× a week) without any contraception in normal physical and psychological condition. It can also defined as inability of a couple to get pregnant to achieve live birth [20]. It’s one of the most common GTB symptoms. Together with endometrial involvement, TB of the Fallopian tubes is the leading cause of infertility in GTB. Prevalence of GTB in infertile population in developing countries is between 5 and 20% and is even higher among patients with tubal factor infertility (39–41%) [17]. There are several hysterosalpingography appearance from tubal involvement such as [17]:

- calcifications showing up as linear streaks;
- tufted tubal outline or tubal diverticula;
- tubal occlusion, especially at the transition between the isthmus and ampulla, multiple occlusion causing a beaded appearance or a rigid pipe stem appearance;
- hydrosalpinx showing up as tubal dilatation with thick mucosal folds;
- peritubal adhesions giving the tube a ‘corkscrew’ appearance, a peritubal halo, or loculated spillage of contrast medium;
- the rare finding of enterotubal fistulae-most common between the sigmoid colon and fallopian tube.

Nowadays, laparoscopy procedure has been done to help physician to diagnose TB of fallopian tubes. It allows better visualization of fallopian tubes, ovaries, and peritoneal cavity and help to restore anatomical abnormalities found in the pelvis if it is possible. There are several findings can be found during laparoscopy which are [16]:

- tubercles on the peritoneal surface;
- inflamed or blue-colored uterus;
- salpingitis, oophoritis, or a tubo-ovarian mass;
- tubal occlusion with hydrosalphinx;
- dye dripping from the fimbrial opening on chromopertubation;
- free peritoneal fluid looking like blood;
- caseation in the pouch of Douglas;
- ‘Frozen pelvis’;
- omental adhesions.

Hysteroscopy should be combined with laparoscopy to exclude/confirmed endometrial involvement. Synechiae or destruction of endometrium by tuberculosis infection should be repaired as soon as possible using estrogen [16].

### Clinical features [16]

Symptoms appears on each patient can be differently found based on severity sites and stage of the disease. Some of the patients may not develop any symptoms. Several symptoms discovered on patients are:

- formation of a large pelvic mass;
- chronic pelvic inflammatory disease symptoms;
- menstrual abnormalities, e.g. Amenorrhea, hypermenorrhea, hypomenorrhea, polymenorrhea, postmenopausal bleeding and uterine abnormal bleeding.
- Excessive vaginal discharge
- General symptoms of typical tuberculosis such as weight loss, anorexia, pyrexia.
- Infertility problems which may be primary or secondary.

## Conclusion

We reported three cases of female with GTB, diagnosed during laparoscopic surgery, confirmed with pathology anatomy and PCR result. A comprehensive history taking, along with correct sampling using various imaging modalities and PCR, will certainly lead to the diagnosis of GTB. It must be noted that infertility due to GTB is irreversible in majority of cases, especially if salpingectomy is achieved promptly. Our case series is a good educational lesson that can be used by obstetrician for better diagnosing and handling of patients with similar problems.

## Abbreviations

TB: tuberculosis
FGTB: female genital tuberculosis
GTB: genital tuberculosis
WHO: World Health Organization
PCR: polymerase chain reaction
BP: base pairs
HE: hematoxylin and eosin
FK UNPAD: Faculty of Medicine, Universitas Padjadjaran
RSHS: Dr. Hasan Sadikin, Hospital
